# Public relations strategies employed by the Ghana Health Service to address COVID-19 vaccine hesitancy: a qualitative inquiry

**DOI:** 10.1186/s41182-023-00519-7

**Published:** 2023-05-12

**Authors:** Ruth Nana Efua McCarthy, Emmanuel Timmy Donkoh, Dominic DeGraft Arthur, Edward Tieru Dassah, Kwame Ofori Boadu, John Ekow Otoo, Ivy Wina Ofori Boadu, Samuel Fosu Gyasi

**Affiliations:** 1 Department of History and Political Studies, Kwame Nkrumah University of Science and Technology, Kumasi, Ghana; 2 Screen and Treat Research Group, Center for Research in Applied Biology, School of Sciences, University of Energy and Natural Resources, UENR, P. O. Box 214, Sunyani, Ghana; 3 Department of Population, Family and Reproductive Health, School of Public Health, Kwame Nkrumah University of Science and Technology, Kumasi, Ghana; 4 Kumasi South Hospital, Kumasi, Ghana; 5 Eastern Regional Health Directorate, Ghana Health Service, Koforidua, Ghana; 6 Department of Medical Diagnostics, Faculty of Allied Health Sciences, Kwame Nkrumah University of Science and Technology, Kumasi, Ghana; 7 Center for Research in Applied Biology, School of Sciences, University of Energy and Natural Resources, UENR, P. O. Box 214, Sunyani, Ghana

**Keywords:** Healthcare workers, Ghana Health Service, Vaccine hesitancy, COVID-19, Public relations

## Abstract

**Background:**

Strategies for developing and advancing good public relations can be recognized in nearly all fields of life without making an exception for the healthcare industry. In the wake of the COVID-19 pandemic, matters of public health have gathered more force. The importance of effective public relations for improving healthcare is highlighted by the position that immediate access to reliable health information should be the hallmark of a just society. However, the strategies available for addressing major threats to the uptake of public health services such as mass vaccination campaigns are not properly studied and documented in the Ghanaian context. This organizational case study explored strategies used by healthcare professionals working with the Ghana Health Service (GHS) to address COVID-19-related vaccine hesitancy in the country.

**Methods:**

We performed a qualitative inquiry with semi-structured in-depth interviews conducted with 25 public health officials of the GHS. The interviews were timed to coincide with the mass deployment of COVID-19 vaccines in four Regions. Participants were recruited through purposive sampling. Data collected included demographic characteristics, perspectives on public relations strategies used in the past year to improve COVID-19 vaccine uptake as well as successes and pitfalls. Thematic analysis was performed with NVIVO software to generate themes from interview transcripts.

**Results:**

Four main themes emerged from the data analysis and these are presented. Healthcare workers perceived vaccine hesitancy to be a threat with the potential to undermine an important strategic organizational goal related to COVID-19 illness. In terms of PR strategies, we determined that a combination of informative, motivational, persuasive and coercive public relations strategies was employed by the Ghana Health Service to address the challenge of vaccine hesitancy. We further found that PR strategies were deployed across both traditional (print, radio, TV) and emerging/social media networks. Officials were optimistic that the strategies would produce results, but were uncertain whether they could attribute current successes or failures to the PR strategies used.

**Conclusion:**

Since the onset of the COVID-19 pandemic, public relations strategies which have been employed by the Ghana Health Service to address vaccine hesitancy are characterized and catalogued. The nature of the audience and PR strategies employed suggests that the effect of these strategies may be short-lived unless they are constantly reinforced by the GHS. These findings show that effective PR strategies exist for addressing vaccine hesitancy in public health practice.

**Supplementary Information:**

The online version contains supplementary material available at 10.1186/s41182-023-00519-7.

## Introduction

The development of vaccines and universal vaccination programmes has been significant in lowering the burden of infectious diseases and the number of related deaths in Ghana [1–3]. In the past, infectious diseases that are now easily preventable were responsible for several deaths [4, 5]. The success of vaccines has led many to downplay the danger posed by emerging infectious diseases and the potential loss of lives in the absence of effective vaccines [6, 7]. The low incidence of confirmed COVID-19 cases and deaths in sub-Saharan Africa has been taken by many for superior natural immunity to SARS-CoV-2 infection and a case against the need for vaccination [6]. There is evidence of growing doubt and negative speculation about the beneficial outcomes and efficacy of vaccination in improving the health of populations [8]. These suspicions have led to vaccine hesitancy [9] and dwindling vaccination coverage [10].

For an immunization programme to work effectively as a public health strategy, healthcare staff must be able to implement it at scale [11, 12]. The mere availability of safe and efficacious vaccines is only one side of the coin; high rates of acceptance and utilization by members of the larger population are critical to driving the intended benefit [12]. Nearly universal vaccination coverage is essential [13], to guarantee protection for individuals, and also for attaining the drastic reduction in transmission that is typically termed “herd immunity” [14]. In order to build more robust and resilient health systems, there is a need to consolidate the best practices from previous pandemics without reinventing the wheel. The devastating impact of the COVID-19 pandemic on healthcare systems with attendant lockdowns and travel restrictions in several African countries focused attention on the work of healthcare organizations.

Ghana endorsed the African Union’s COVID-19 Vaccine Development and Access Strategy to vaccinate at least 60% of the population of member states with a safe and efficacious vaccine by end of 2022, to achieve the population-level immunity needed to bring the pandemic under control [6]. In the pursuit of this target, six vaccines have been approved and deployed in Ghana through the Health Service: these are (1) AZD1222 Covishield (Oxford/AstraZeneca); (2) ChAdOx1 nCoV-19 Vaxzevria (Oxford/AstraZeneca); (3) JNJ-78436735 Janssen (Johnson & Johnson), (4) Gam-COVID-Vac Sputnik V (Gamaleya); (5) BNT162b2 Comirnaty (Pfizer/BioNTech); (6) mRNA-1273 Spikevax (Moderna), with the AstraZeneca/Oxford vaccine being the most widely deployed [9].

Available data on public attitudes toward vaccines indicate substantial vaccine complacency [15]. Unlike many other pharmacological interventions, public confidence in vaccination is at an all-time low following the COVID-19 pandemic; influenced by unhealthy suspicion of the safety and effectiveness of vaccines, scepticism about the interests of health workers and healthcare organizations and their products, and of the policies of the scientific establishment [8, 16]. At the peak of the COVID-19 pandemic, efforts by governments around the world to rapidly fund, develop and distribute vaccines with emergency use authorization from the World Health Organization (WHO) led to fears about vaccine quality and safety [17]. Aversion to vaccines may be mediated by health beliefs [8]. Ironically, the far-reaching success of vaccination may in itself be a catalyst for anti-vaccine sentiment by lowering the perception of disease risk and severity. Factors sustaining aversion to COVID-19 vaccines resemble well-documented general anti-vaccine sentiment [18]. Broadly, these can be classified into vaccine-related attributes, vaccine-related attitudes and beliefs, and the political environment [19]. To overcome this scenario, a multi-pronged evidence-based approach is required to instigate behaviour change and address vaccine hesitancy. Undoubtedly, the best examples of public relations (PR) need to be considered by healthcare organizations to leverage credible and transparent political leadership in order to drive a robust agenda of health delivery to address the “infodemic” fueling vaccine hesitancy [16, 20].

Public relations practice involves a deliberate and continuous effort to create and sustain mutual understanding and goodwill between an organization and its publics [21]. PR practice helps organizations to manage their reputation by identifying public views of the organization and by taking steps to shape this with strategies that maintain a positive feeling about organizational goals [21]. Hazleton and Long’s public relations process model show that the objectives of public relations can often be seen in and achieved through the impact of well-crafted communication with target audiences if the audience can accurately decode the intended meaning of the messages [22]. A taxonomy of public relations strategies in use by organizations has been compiled based on change planning, models of PR and the psychological functions of messages [23, 24]. This taxonomy recognizes that PR strategies may be characterized as (1) informative, (2) facilitative, (3) persuasive, (4) coercive, (5) bargaining, and (6) cooperative [25].

Before the COVID-19 pandemic, the Ghana Health Service had successfully implemented mass vaccination campaigns for yellow fever and polio in adults in addition to the largely successful infant immunization programme [7]. Vaccination against human papillomaviruses has been conducted in four districts from 2013 to 2015 among adolescent girls in a three-dose schedule with a quadrivalent vaccine [26]. Valuable lessons learnt from these previous vaccination campaigns have been documented and were applied in the deployment of vaccines for COVID-19 in the country. By leveraging existing vaccination and health systems rather than configuring new ones, healthcare professionals in the country were able to deploy COVID-19 vaccines within a few months with existing infrastructure and less than 5% vaccine losses due to expiry despite the short shelf-life of vaccines on hand. Despite these successes, the full story regarding PR strategies employed to avert vaccine hesitancy as a threat to organizational goals remains unreported. Indeed, previously published work reported on PR tools such as social media, and print media among others [16, 20]. This is the first work to report on PR strategies based on Hazleton and Long’s (1988) public relations process model. We sought to explore and document PR strategies used by healthcare professionals to address COVID-19-related vaccine hesitancy in the country using an established PR process model to inform public health policy, practice and research.

## Materials and methods

### Study design and setting

As the PR strategies employed by public health officers of the GHS to promote vaccine compliance in the Ghanaian context has yet to be documented, a qualitative research method designed as a case study was embarked. The study was conducted among public health officials of the Ghana Health Service (GHS) from the Bono and Eastern Regions of Ghana from November 2021 to February 2022 after the initial mass immunization campaign and then in the Ahafo and Ashanti Regions after a more expanded national immunization campaign from March to June 2022.

For context, the first cases of COVID-19 in Ghana were detected among immigrants and officially announced on 12 March 2020 by the Minister for Health [27]. From the end of March 2020, the Government imposed partial lockdowns in the two biggest cities, Accra and Kumasi, which were known epicentres for the pandemic, initially spanning three weeks. Known contacts of infected persons continued to be traced and quarantined. As the pandemic raged in other cities across the country, initial measures were followed by more stringent ones in subsequent weeks. From July 2020, some of the restrictions were eased slowly, including the opening of the airports and land borders from 1 September 2020. By 3 January 2021, the Government of Ghana announced the re-opening of academic institutions to allow all students to go back to school but restrictions on nightclubs and beaches were still in place [27].

Ghana received the first shipment of 600 000 COVID-19 vaccinations from the COVAX facility on 24 February, 2021 [28]. This consignment paved way for an initial vaccination campaign, which commenced on 1st March 2021 following the vaccination of the President and was focused on health workers, high-risk individuals, and front-line personnel living in Greater Accra, Ashanti and parts of Central regions to receive their first dose of the COVID-19 vaccine. This initial consignment was supplemented by a further 350 000 doses on 7 May, 2021 [29]. The initial success of these measures led to an easing of restrictions on movement: people were able to move much more freely between cities again. However, the case count would subside before escalating again during the end-of-year holidays in 2021. To improve COVID-19 vaccine uptake after the first mass vaccination campaign in December 2021 failed to prevent a new wave of infections, the Ministry of Health instituted its first National COVID-19 Vaccination Day in February 2022. Since then, the GHS has been able to conduct mass community vaccination campaigns all over the country in addition to providing continuous access to vaccines at designated health facilities [30]. Out of the 20 million individuals targeted for vaccination in Ghana, approximately 12 million have received at least one dose of vaccine, 9 million have received the last dose of the primary vaccination series and a further 2 million have received a booster dose as of March 2023.

The GHS is an administratively autonomous organization established by law (Act 525 of 1996) sixteen and is vested with the authority to implement national policies formulated by the Ministry of Health (MOH) through the governing board, the Ghana Health Service Council [31]. Following the reorganization of the MOH and healthcare administration in the country, it was recognized at the time that the growing level of managerial responsibility that was delegated to district hospitals required a new organizational paradigm to oversee. The formation of the GHS comes as one of the vital strategies recognized by the Health Sector Reform of the 1990s, which was guided by the Medium-Term Health Strategy [31] in the pursuit of establishing a more equitable, efficient, accessible and responsive healthcare system.

The GHS is the sole implementing authority of health policy in Ghana by providing and managing comprehensive and accessible health services with special attention to primary health care at regional, district and sub-district levels. Promoting health, healthy living and good health habits by people is seen as a key function. The GHS has sixteen regional health directorates, with one in each of the administrative regions of the country. Each region also has district health directorates [31]. The PR responsibilities of the GHS are a function of the office of the Director General. However, as a result of the stratified nature of health administration, district and regional directors of health also play a significant role in engaging the public.

### Participants and recruitment

Key informants or health officials with at least 3 months of public health advocacy experience related to COVID-19 vaccination were purposively approached to provide feedback relevant to the phenomenon under study [32, 33]. Interns and contract/temporary staff were excluded from the study. At the regional and district offices of the GHS in the Bono, Ahafo, Ashanti and Eastern Regions, the general purpose of the study was discussed with a large group of selected officials and those who consented were scheduled for interviews. High-ranking officials from the regions were purposively sought for interaction. Selected officials or key informants were provided information about the rationale for the study and issues regarding the ethical conduct of research such as voluntary participation, potential risks and respect for privacy when reporting all findings were agreed upon in advance. Interviews lasting approximately 30 to 45 minutes were conducted in-person as scheduled until data saturation was attained. Data saturation occurred when no new themes and sub-themes emerged after conducting three consecutive interviews.

### Data collection

Data for the study were collected from November 2021 to June 2022. Participants were contacted in advance via telephone calls and text messages to remind them and confirm all interview arrangements. Participants were also required to complete a short online survey to provide demographic metadata. Informed consent (including permission to digitally record the interview session) was documented before in-depth one-to-one interviews with public health managers and administrators were conducted by a researcher trained in qualitative research.

All interviews were opened with an exchange of pleasantries and general conversation to create rapport and to set respondents at ease to give feedback without inhibition or apprehension. Assurances were also given regarding the confidentiality of conversations and the participant’s right to anonymity. This created a congenial environment for data collection with the aid of a semi-structured interview guide (Additional file 1: S1 Appendix A.docx: Interview guide for in-depth interviews). Participants’ demographic data including gender, official designation and years of work-related experience were captured.

### Research tool/instrument

The study relied on a semi-structured interview guide to elicit information about (1) participants’ knowledge of the threat of vaccine hesitancy to the achievement of organizational goals; (2) PR strategies employed by the organization to improve attitudes toward vaccinations; (3) the impact of available PR options; (4) contextual factors that promote the success or failure of these PR strategies. A consultative approach was followed to develop the instrument based on empirical literature and expert guidance. The instrument was pretested in a healthcare organization to elicit feedback on test reliability and validity for improvement. The interview guide can be found as a supplementary file (Additional file 1: S1 Appendix A.docx: Interview guide for in-depth interviews).

### Data analyses and rigour

Data collected from in-depth interviews were subjected to the thematic analysis procedure to discover public relations strategies embedded within the routine health promotion practices of public health officials [34, 35]. Data coding was accomplished in four separate stages [36] as follows: theme structuring, coding using NVIVO software version 10.0.638.0 (QSR International, MA, USA), organization of themes, and interpretation of thematic data. The approach was useful for conducting a thorough analysis of the data in search of meaning [37]. All coding of the data was done in duplicate in NVIVO and nodes or aggregates inductively plotted into potential themes. Data coding was performed concurrently with interviews by a trained postgraduate student and another member of the research team with publication experience in qualitative research. In addition, all transcripts and codebooks were reviewed by an experienced faculty in the capacity of project supervisor. The supervisor acting as an “auditor” ensured that the data obtained from coding were reliable by confirming intercoder variability to be within acceptable margins [38]. The codes were categorized and subjected to a thematicization process by the research team made up of experts in qualitative research and health service delivery. Potential themes were discussed and refined in a series of team meetings until they were organized by group consensus [34].

In the final stage, the data were assessed for trustworthiness based on reasonable evidence of credibility, transferability, dependability, and confirmability [39, 40]. Credibility examines the level to which the research results reflect reality and is representative of the participants' views [39, 41]. The principal investigator checked with participants to fully understand the ideas represented and adequately captured participants' storylines to ensure credibility. Dependability refers to the quality of the integrated processes of data collection, data analysis, and theory generation that can be audited [41]. This was achieved by a detailed description of the research methodology (recruitment process, data collection, data analysis) in line with the consolidated criteria for reporting qualitative research (COREQ) reporting guidelines [40, 42]. Transferability refers to how the study results could be used in different areas and contexts [40]. To ensure this, a comprehensive description of the study setting was provided. Confirmability was ensured by keeping records of the field notes and voice records.

### Ethical issues

The study was approved in September, 2021 by the Committee for Human Research and Ethics, the Institutional Review Board of the University of Energy and Natural Resources (CHRE/CA/046/21). The study was conducted in conformity with the requirements of ethical propriety: all participants were adequately informed about the study before indicating their desire to participate in recorded interactions and also provided written voluntary consent for recorded data to be anonymized prior to dissemination.

## Results

Altogether, 25 officials of the GHS comprising 10 directors/managers of health service and 15 disease control/public health officers granted interviews for the study. These individuals were aged between 36 and 69 years. The number of years of participants’ experience in active service ranged from 2 to 10 years and all participants had obtained a minimum of undergraduate education.

Four main themes emerged from the data analysis and these are represented in Table 1. These are a) organizational goals and potential threats; b) knowledge of public relations strategies in use by organization c) tools/media employed for the realization of public relations strategies d) anticipated impact of PR strategies. In addition to the main themes, a number of sub-themes were synthesized.

**Table 1:** Summary of themes and sub-themes from the transcribed data

Themes	Sub-themes
Organizational goals and potential threats	1. Perception of vaccine hesitancy 2. Drivers of vaccine hesitancy
Public relations strategies in use by organization	1. Informative PR strategy 2. Persuasive PR strategy 3. Facilitative PR strategy 4. Coercive PR strategy
Tools/media employed for the realization of public relations strategies	1. Traditional media 2. New/social media 3. Facilities
Anticipated impact of PR strategies	1. Positive impact 2. Scepticism 3. Contextual factors

*Source:* Fieldwork

*PR:* public relations

### The perceived threat of vaccine misinformation to the achievement of organizational goals

Vaccination among the public was considered to be an important public health target. As the organization was in pursuit of a threshold phenomenon described by participants in technical language as “herd immunity”, most participants considered this goal of their organization to be somewhat under threat insofar as large aspects of the public remained sceptical about the judicious and safe use of the vaccines.

*Yes. Covid 19 is easily spread from one person to another. With the vaccine, the transmission rate can be reduced so that the vulnerable will be protected (that's the whole point of herd immunity) (Akwesi; Disease Control Officer, ER)*

Yes, Vaccine hesitancy is very much present among the general populace and this can adversely affect targets, hinder the achievement of herd immunity and increase the chances of being infected *[with]* complications *(Veronica*, *Medical* *Superintendent,* *BR)*

However, there were various obstacles to be overcome to successfully obtain this organizational goal. One such threat was non-adherence to vaccine advice. In terms of how hesitancy constituted a threat, respondents anticipated that negative sentiments could only delay the attainment of herd immunity but not ultimately prevent it.

*Yes,* *vaccine* *hesitancy* *is* *a* *threat* *to* *the* *vaccination* *program.* *People* *refusing* *to* *vaccinate* *will* *prevent* *the* *achievement* *of* *the* *coverage* *that* *will* *enable* *herd* *immunity* *and* *therefore* *the* *full* *benefit* *of* *vaccination* *to* *the* *communities* *and* *the* *country* *in* *general* *(Ephraim*; *Health* *Promotion* *Officer,* *AhR)*

*Yes.* *It* *will* *lead* *to* *delays* *in* *achieving* *herd* immunity *(Assumaning;* *Medical* *Superintendent,* *AR)*

*…any obstruction to increasing vaccine acceptance and coverage slows us down in attaining these targets* *(Diana;* *Public* *Health* *Manager,* *AR)*

Several reasons could be attributed to the threat of hesitancy such as religious persuasion, misinformation, lack of proper orientation regarding the scientific basis of vaccines and the result of widespread misinformation across multiple social media channels.

*…this* *is* *a* *result* *of* *myths* *and* *misconceptions* *about* *the* *side* *effects* *of* *vaccines* *and* *other* *religious* *beliefs* (Siriboe; *Health* *Promotion* *Officer,* *AR)*

*…this* *is* *because* *people* *have* *a* *whole* *lot* *of* *negative* *ideas* *and* *philosophies* *about* *this* *vaccine*… *(Fred;* *Medical* *Superintendent,* *BR)*

*Vaccine* *hesitancy* *is* *strongly* *influenced* *by* *misinformation...conspiracy* *theories* *over* *social* *media* *about* *the* *COVID* *vaccines* *are* *the* *main* *cause* *(Millicent,* *Hospital* *Administrator,* *AhR)*

Despite the perceived threat of vaccine hesitancy to the organization’s goals, this was more of a perception than the prevalent reality. This compelled some respondents to believe that vaccine hesitancy was not a threat at all to the organization’s ambitions. Indeed, during the study period, most countries around the world were grappling with shortages of vaccine doses which was seen as a bigger threat than hesitancy.

No. Vaccine hesitancy is not primarily an issue. Most patients request for [the vaccine] voluntarily. The problem is the availability *(Amelor;* *Disease* *Control* *Officer,* *AhR)*

However, participants agreed that these threats were of such a nature that they could be addressed with an effective PR strategy. Respondents reported coming across individuals with beliefs ranging from the very entrenched to mere ‘hearsay’ about the harmful nature of vaccines. It was considered that to some extent, there was a real possibility that negative beliefs could be dispelled by offering accurate scientific information through credible officials and agents.

Vaccine hesitancy though can affect the expected target: [however,] the challenge can be addressed by public health personnel using the right approach and the best information *(Abenaa,* *Public* *Health* *Manager,* *BR)*

*This* *is* *because* *people* *have* *a* *whole* *lot* *of* *negative* *ideas* *and* *philosophies* *about* *this* *vaccine.* [Effective] PR work will help explain the issues better and also *improve* *public* *sentiment* so that *more* *people* will be willing to take the vaccine *(Fred;* *Medical* *Superintendent,* *BR)*

*Vaccine* *hesitancy* *is* *strongly* *influenced* *by* *misinformation...conspiracy* *theories* *over* *social* *media* *about* *the* *COVID* *vaccines* *are* *the* *main* *cause* *(Millicent,* *Hospital* *Administrator,* *AR)*

The expert interviews also revealed that health workers felt the need for their organization to promote PR practice and to engage the public by confronting the new wave of misinformation and adapting to the speed of information spread through new and emerging media while maintaining media sources that were already seen as more credible because of a positive public image built over time.

The EPI [Expanded Programme on Immunization] *was* *successful* *because* *of* *PR.* *However,* *that* *was* *easier* *in* *those* *times* *because* *most* *information* *came* *from* *National* *TV* *and* *radio* *stations.* *So,* it is an issue of adapting PR strategies to modern times *(Veronica,* *Medical* *Superintendent,* *BR)*

*A* *target-*specific public relations strategy can help dispel some of the myths peddled *by* *anti-vaccine* *campaigners* *and* *promote* *more* *detailed* *health* *education* *on* *the* *advantages* *of* *vaccines* *(David,* *Administrator,* *ER)*

### Public relations strategies used by the GHS to promote vaccine acceptance

Since the start of the pandemic, public health managers at the GHS have devised various strategies to engage their publics and generate sympathy towards their organization’s objectives. The present study identified themes reflecting elements of PR strategy embedded within the messages encoded by officials of the service and broadcast to the public. The elements of PR strategy discovered from interactions with key personnel of the GHS are outlined.

### Informative PR strategy

The service proactively employed a communication strategy to make as much public information available as possible through the health workforce [43]. Health workers received regular training spearheaded by national, regional and district-level Public Health Emergency Management Committees. Also, regular press briefings were held by the Presidential Task Force on COVID-19 which was supported by the GHS Headquarters led by the Director General. After each press briefing, the Minister of Health followed up with a meet-the-press session to elaborate on pertinent issues that needed clarification. The “open door” strategy where journalists could ask questions and get feedback from the Minister of Health and the leadership of the Health Service encouraged radio and television stations nationwide to dedicate airtime to broadcast the briefing sessions and helped the GHS to set the tone for public discussion with authoritative information throughout the COVID-19 pandemic [43]. In turn, broadcast media outlets would call previously-coached health workers to accentuate health-related information and further explain concepts in simple terms to the listening audience in a win–win scenario for media outlets seeking content and health officers implementing an informative PR strategy.

We sent our men to the radio stations to educate the public on the dangers of refusing to accept the vaccines. We told them to stress the need for radio stations to talk to the experts. You know charlatans try to take advantage of people by posing as knowledgeable individuals. I think to some extent the strategy worked *(Judith,* *Public* *Health* *Manager,* *BR)*

From the early days of the COVID-19 pandemic, the GHS relied on electronic and print media to provide education to people on the importance of taking the vaccine. Through the Disease Control Unit, the GHS produced and circulated informative educational material [44]. Some officials noted:

At almost all of our workshops, the need for more education was constantly stressed. We on the inside had access to vital information from the WHO: we attended webinars and several zoom meetings. But what about the populace? Who was organizing webinars for them? So, we felt that we needed to put out as much education as possible *(Siriboe,* *Health* *Promotion* *Officer,* *AR)*

We always felt that education was our best strategy to fight this [epidemic of misinformation]. *Basically,* *some* *informative* *flyers* were produced and circulated to our facilities, marketplaces, and social media *(Temaa,* *Disease* *Control* *Officer,* *ER)*

Additionally, a COVID-19 dashboard was launched to provide near real-time information about the rising number of cases of COVID-19. Throughout the pandemic, this dashboard was considered the most authoritative source of information and was mostly relied upon during official briefings.

*I* *heard* *many* *conspiracy* *theories* *about* *Governments* *falsifying* *case* *numbers* *to* *impose* *lockdowns.* *But* *I* *think* *that* *here* *[in* *Ghana],* *the* *figures* *we* *put* *out* *on* *the* *dashboard* *have* *helped* *a* *lot…* *(Amelor,* *Disease* *Control* *Officer,* *AhR).*

### Facilitative PR strategy

The GHS disseminated vaccine-related communication in simple language to create awareness about vaccination schedules, sites and protocols [44]. These infographics were meant to be circulated widely on social media pages to provide reliable and credible official information about vaccination schedules and thereby facilitate the uptake of vaccines by citizens.

If you want the people to buy into the exercise, you don’t want them to be frustrated by a lack of information that allows them to act independently… these are traders, office workers and generally busy people… We situated vaccination sites close to them and we tried to communicate that ahead of time *(Williams,* *Pharmacist,* *AR)*

### Persuasive PR strategy

Another public strategy employed by the GHS was the use of press conferences with messaging calculatedly crafted to appeal to the public’s values and emotions and to instigate public sentiment for vaccination.

*One* *of* *the* *things* *that* *we* *also* *did* *was* *to* *engage* *high-profile* *individuals* *and* *empower* *them* *to* *spearhead* *our* *communication.* *In* *this* *strategy,* *the* *President* *of* *Ghana* *readily* *comes* *to* *mind.* *In* *fact,* this strategy was so useful it attracted international commendation *(Akwesi;* *Disease* *Control* *Officer,* *ER)*

*The* *use* *of* *opinion* *leaders,* *organized* *groups,* *and* *influential* *persons* *to* *champion* *the* *course* *of* *the* *COVID-19* *vaccination* *was* *a* *vital* *strategy* *for* *us* *in* *addition* *to* *the* *use* *of* *social* *mobilization* *and* *risk* *communication* *messages* *to* *increase* *the* *uptake* *of* *the* *COVID-19* *vaccination* *(Ephraim,* *Health* *Promotion* *Officer,* *AhR)*

The service also arranged for the first dose of vaccines in the country to be given to prominent chiefs, political figures and prominent civil society actors to persuade the citizens of their safety and avoid hesitation.

### Coercive PR strategy

A coercive strategy was also employed by the Service. Although no vaccine mandates have been issued in Ghana at the time of this report, this does not mean that it has escaped officials from the Health Service. Repeatedly, high-ranking officials were captured on record attempting to convince members of the public through messages that hint at the imminent imposition of restrictions at border posts and social gatherings.

Before the Christmas festivities, we secured enough vaccines for the population. We wanted as many people as possible to get the jab. Therefore, *I* *am* *not* *surprised* *that* *there* *were* *hints* *that* *without* *getting* *vaccinated,* *for* *instance,* you can’t attend public events *(Diana,* *Public* *Health* *Manager,* *AR)*

More commonly, the coercive strategy has been used by officials of the GHS when referring to travel requirements for passengers to produce evidence of a negative polymerase chain reaction (PCR) test result within 48–72 hour of boarding at international airports.

### Motivational PR strategy

To maintain a positive internal sentiment about vaccination within the organization, the GHS employed various communication strategies to keep health workers motivated. Health workers were depicted in several ways as “front-line”, “at-risk”, “heroes” and the like to keep them as motivated as possible. These adjectives were meant to court public sympathy, promote organizational values and ultimately prevent and reduce hesitation about vaccination. In addition to motivating language descriptors, health workers received regular testing services at no cost, donations of personal protective equipment, off days, priority access to available vaccine doses [28, 29] and income tax waivers from the state for almost a year for their work in helping to promote health. Although there were initial implementation challenges such as identifying those who qualified as front-line workers, delays and non-uniform roll-out across all categories of health workers, the measures were constantly mentioned during regular press briefings organized by the Service and the Presidency to improve health worker morale.

*You* *know* *[pause]* *healthcare* *workers* *are* *role* *models* *for* *friends,* *family* *and* *society* *at* *large.* *We* *all* *saw* *viral* *videos* *of* *healthcare* *workers* *in* *other* *countries,* *and* *even* *in* *Ghana,* *calling* *on* *the* *public* *to* *abstain* *from* *COVID-19* *vaccinations.* *So,* *from* *the* *very* *start* *steps* *were* *taken* *to* *motivate* *our* *staff* *to* *step* *forward* *to* *be* *vaccinated* *and* *encourage* *others* *to* *do* *same* *(Veronica,* *Medical* *Superintendent,* *BR)*

### Tools/media employed for the realization of public relations strategies

Tools employed by health officials for the realization of organizational goals related to vaccines ranged from stakeholder engagements from the planning to the implementation stages, public education on COVID-19 vaccination through community durbars and use of community information centres,  the establishment of mobile vaccination posts at health facilities and vantage points, advocacy meetings with opinion leaders and social groups and public health emergency committee meetings to provide feedback on all COVID-19 interventions.

*We* *can* *also* *talk* *about* *i*nfographics on TV, on social media, and in *hospitals,* all these tools were employed to drive strategic messages *(Judith,* *Public* *Health* *Manager,* *BR)*

#### Public engagement through traditional media

Traditional media channels were seen as important outlets by officials of the GHS for the dissemination of information and public engagement. Both national and private radio and television channels were used to broadcast press briefings, interviews, and soundbites and generally mobilize community members.

*Some* *of* *the* *strategies [tools]* *employed* *by* *the* *GHS* *include* *1.* *Continuous* *social* *mobilization* *using* *different* *media* *outlets* *to* *educate* *people* *on* *the* *vaccination* *process* *and* *the* *benefits* *thereof.* *2.* *Continuous* *press* *releases* on the epidemiology of the *COVID-19* *outbreak* *is* *one* *strategy* *that* *is* *to* *help* *with* *vaccine* *hesitancy* *3.* *Use* *of* *role* *models* *in* *society* *such* *as* *the* *president*, *vice* *president* *and* *their* *spouses* *to* *take* *the* *jab* *on* *national* *television,* *among* *others.* *(Fred,* *Medical* *Superintendent.,* *BR)*

Participants felt that although the presence of the GHS on social media was a show of the Service’s willingness to evolve to keep up with the changing demands of the times, the traditional media such as radio and television was still essential for generating the right public sentiment because of the tendency for social media to be used to carry misleading information orchestrated by pranksters and other groups opposed to vaccination and other medical products.

#### Public engagement through new and emerging media

Nevertheless, the GHS also embraced new and emerging channels such as dashboard websites, Instagram, Twitter and Facebook to promote communication targeted at improving vaccine behaviour. These channels conveyed communication crafted with the aforementioned PR strategies to a wide range of audiences who may otherwise be missed by traditional media such as adolescents and young adults.

*First* *of* *all,* *the* *Ghana* *Health* *Service* *has* *a* *dedicated* *website* *where* *all* *official* *information* *concerning* *COVID-19* and *v*accines *is* *found.* *A* *COVID-19* *helpline* *was* *announced* *last* *year* *as* *well:* *this* *is* *a* *24-*hour dedicated line that people can access to have a professional discuss their misgivings about the vaccines. *(Akwesi;* *Disease* *Control* *Officer,* *ER)*

#### Clinic/facility engagements

As a complementary measure, the GHS also engaged members of the public who visited healthcare facilities with regular educative campaigns at outpatient departments (OPDs). These interactions between health workers and facility patrons could offer several advantages.

Forums are organized where the general public is allowed to ask questions and share their experiences related to the COVID vaccine and the vaccination process. *(Williams,* *Pharmacist,* *AR)*

*Education* *and* *testimonials* *are* *major* *features* *of* *our* *PR* *strategy.* *So,* *we* *do* *regular* *education* *at* *our* *OPD* *to* *sensitize* *people* *about* *vaccinations* *and* *to* *diffuse* *common* *myths.* *Also,* *testimonials* *of* *people* *who* *have* *taken* *it* *are* *brought,* *especially* *those* *who* *are* *popular* *and* *have* *integrity,* *so* *that* *others* *will* *know* *there* *is* *nothing* *to* *fear.* *(Millicent,* *Administrator,* *AhR)*

### Expected impact of PR strategies

The impact of the public relations strategy employed by the GHS can be described as positive overall. Participants felt strongly that the strategies implemented by the Service could play an important role in preempting crisis levels of hesitancy by making more information available and evoking a cooperative public disposition. A number of officials felt that the GHS was winning the information war with readily accessible credible information.

*People* *now* *have* *information* *that* *wasn't* *available* *initially.* *All* *the* *unanswered* *questions* *about* *vaccines* *are* *being* *handled.* *(Judith,* *Public* *Health* *Manager,* *BR)*

*When* *there's* *an* *official* *website* *or* *credible* *source,* *we* *can* *resort* *to* *resolving* *conspiracies* *and* *arguments* *with* *information* *from* *these* *places.* *(David*, Administrator, *ER)*

In areas where vaccine uptake was high officials were eager to attribute the positive public attitude towards vaccination to the impact of effective PR practice and education. However, some participants were careful not to ascribe much success to the PR strategies employed citing entrenched beliefs and the potential for complacency on the part of agents of the organization.

*It* *has* *been* *positive* *because* *the* *application* *of* *the* *above* *PR* *[strategies]* *has* led to an increase in the uptake of the vaccines and people are more committed to taking the vaccines *(Abenaa,* *Public* *Health* *Manager,* *BR)*

*The* *above-listed* *PR* *strategies* *have* helped [to] *educate* *the* *people,* *cleared* *misconceptions* *and* *sustained* *confidence* *in* *COVID* *vaccination.* *(Fred,* *Medical* *Superintendent,* *BR)*

Not much. From my personal view, it's only when close contacts to an individual who is hesitant contract the disease that perceptions change, no matter the PR strategy used. *(Amelor,* *Disease* *Control* *Officer,* *AhR)*

*The* *response* *has* *been* *good* *so* *far.* *Just* *that* *there* *is* *more* *work* *to* *be* *done.* *(Williams,* *Pharmacist,* *AR)*

### Contextual factors responsible for the success or failure of PR strategies

Participants gave their impressions about some contextual factors that in their opinion could promote or undermine the PR strategies discovered in the work. Key contextual factors that were identified to be relevant for the success of organizational PR regarding the threat of vaccine hesitancy were the need to build relationships of trust with community members over time and to recruit influential community figures to not only carry information to the community but also to mediate the relationship between the community and the organization. These influential persons are typically religious leaders, opinion leaders, market queens and elected representatives at the district assembly.

Strong social networks in the communities and trust in influential *persons* [can make *all* *the* *difference].* *(Ephraim,* *Health* *Promotion* *Officer,* *AhR)*

*…*the success of any *PR* *strategy* *depends* *on* *how* *well-informed* *the* *PR* *person* *is* *and* *how* *well* *the* *information* *available* *to* *him* *is* *being* *delivered* *to* *the* *audience.* *However,* *if* *the* *information* *is* *not* *well* *delivered,* *the* *strategy* *will* *fail* *(Assumaning,* *Medical* *Superintendent,* *AR).*

A key thing is trust. You can provide information but people will only take that information if they deem the source trustworthy. Some people do not trust the big pharmaceutical companies and will not take any information from them as credible. The population must be studied to know the *sources* *they* *are* *most* *likely* *to* *trust.* *PR* *can* *then* *use* *these* *agencies* *to* *promote* *vaccine* *acceptance.* *In* *Ghana,* *for* *example,* most of the Christian *faith* are more likely to accept information from their church leadership than the Government. *(Mansa,* *Medical* *Superintendent.* *ER)*

However, certain pitfalls were discovered that must be taken into consideration when designing PR programmes. At the height of the pandemic and during the several months after the onset of the mass vaccination campaigns, several challenges faced by the country’s health and political leadership in securing vaccines from donors on schedule were documented by television and print media. As a consequence, healthcare workers were anxious that there could be shortages or that promises to deliver vaccines could not be honoured. Public health officials designing credible PR strategies need to bear this in mind and adopt strategies for unexpected circumstances such as vaccine shortages from supply chain shocks and vaccine nationalism. In addition, respondents were of the impression that programmes must consider the high rates of illiteracy in rural areas and any potential conflicts with religious beliefs which may ensue from conservative radical teaching. Respondents cited information from some religious outlets to the effect that human bodies are sacred entities and urged adherents to view vaccination exercises as an artificial manipulation that amounted to an act of desecration and had eternal consequences for the adherents’ soul. Also, health officials were aware of religious teaching that viewed vaccination exercises as covert birth-control strategies and consequently encouraged adherents to abstain from all forms of vaccination campaigns.

*However,* *failure* *will* *stem* *from* *frequent* *shortages* *of* *vaccines* *and* *a* *lack* *of* *political* *will* *to* *implement* *programmes* *related* *to* *vaccines.* *(Assumaning,* *Medical* *Superintendent.* *AR)*

*I* *will* *tell* *you* *about* *an* *incident* *that* *shocked* *all* *of* *us* *here.* *When* *school* *children* *returned* *to* *school* *after* *the* *COVID-19* *hiatus,* *several* *school* *visits* *were* *planned…As* *soon* *as* *we* *parked* *the* *Service’s* *vehicle* *on* *the* *school* *compound,* *all* *the* *children* *trooped* *out* *and* *headed* *home.* *This* *was* *during* *the* *peak* *of* *debates* *about* *a* *team* *of* *French* *scientists* *purported* *to* *be* *planning* *vaccine* *trials* *in* *Africa.* *We* *had* *to* *go* *into* *the* *community* *to* *reassure* *the* *parents* *that* *no* *vaccination* *was* *planned.* *(Diana,* *Public* *Health* *Manager,* *AR)*

A key lesson learnt here by the team was the importance of community leaders in the planning of even routine public health exercises. In the wake of this incident, the district health team was able to gain the confidence of community leaders after they were thoroughly assured that no vaccination was planned.

*“There* *was* *an* *occurrence* *where* *a* *person* *shared* *that* *they* *experienced* *an* *adverse* *event* *following* *vaccination* *(AEFI);* *within* *that* *period,* *the* *average* *number* *of* *people* *who* *came* *in* *for* *the* *vaccine* *decreased.* *Notwithstanding,* *it* *is* *observed* *that* *most* *people,* *having* *heard* *that* *someone* *they* *knew* *took* *the* *vaccine* *had* *the* *motivation* *to* *take* *the* *vaccine* *as* *well…we* *must* *get* *those* *who* *the* *people* *listen* *to* *so* *they* *get* *them* *to* *change.”* *(Veronica,* *Medical* *Superintendent,* *BR)*

The decentralized administrative structure of the Ghana Health Service allowed top-level officials to orchestrate a PR campaign that was nationwide in scope and relevant to local contexts at the same time. This was achieved by broadcasting pre-recorded messages in the local dialect; on the radio for the cities and towns and on megaphones at the community level.

## Discussion

The present work explored strategies used by healthcare professionals working with the GHS to address COVID-19-related vaccine hesitancy in the country through in-depth interviews with key informants. A thematic analysis of the informant interviews revealed that healthcare workers perceive vaccine hesitancy to be a threat with the potential to undermine the strategic goals of their organization especially related to the attainment of herd immunity against COVID-19. Using the Hazleton and Long's (1988) public relations process model [22] and a taxonomy of PR strategies based on change planning [24], models of PR and the psychological functions of messages [23], we discovered that a number of PR strategies including informative, facilitative, persuasive, coercive and motivational PR strategies were employed to diffuse this threat across both traditional and emerging media networks.

Deterioration of public trust in vaccination, in general, has been reported by many authors in line with the present study [17]. General suspicion of vaccination, misunderstandings about infection severity, and an aversion to medical innovation are known to propel vaccine-hesitant behaviour and even non-compliance to public health measures such as the wearing of nose masks [45]. The consensus was that although the concept of herd immunity was an important one and had been communicated as an important organizational goal in the fight against the COVID-19 pandemic by credible and authoritative sources and therefore was well assimilated and aspired to, the communication had not been unambiguously passed on to the general public who in turn had conflicting explanations about how this goal could be achieved. Healthcare workers interviewed spoke vaguely about organizational targets for herd immunity without referring to any recognizable technical indicators.

Healthcare workers are expected to play a significant role in addressing commonly held misconceptions and driving widespread acceptance of vaccine products among the public [46]. The Centers for Disease Control and Prevention (CDC) highlights 12 strategies to promote vaccine confidence and uptake. These include vaccine ambassadors, medical provider vaccine standardization, medical reminders, motivational interviewing, financial incentives, school-based vaccination programmes, home-delivered vaccinations, workplace vaccination, effective messaging by trusted messengers, provider recommendation and combating misinformation [47]. Consistent with this position, officials of the GHS demonstrated an awareness of their role in the fight against misinformation and maintaining positive vaccine behaviour among members of the public [30].

The role of a health advocate requires employees to develop an awareness of key organizational goals and to be able to work towards achieving them. Here, this was done through various PR strategies and communication tools. Strategies commonly used involved information dissemination, facilitation of desired behaviour, persuasion, coercion, and motivation [48]. Data from several recent studies on COVID-19 vaccine hesitancy in Ghana point to the effectiveness of these strategies employed by the GHS before and during the mass roll-out of vaccines from December 2021 [9, 49–51].

Organizations typically use the informative strategy to advance objective facts about situations with the assumption of well-educated and self-motivated publics [22, 23, 52]. Without necessarily making conclusions or advancing opinions, this strategy succeeds because the intended audience will derive the expected pattern of behaviour by making the right conclusions from available data provided it is accurate. A key function of this strategy is providing public education about subjects that inform or are hinged upon organizational goals [52]. In the case under study, we see the GHS advancing knowledge about the vaccine manufacturing process, the relevance and conduct of trials and in general even about public health concepts to counter anti-vaccine campaigns on social media [30].

Educative campaigns may offer new or alternative solutions to existing threats to an organization’s interests such as an under-informed audience. Additionally, they may stand out by the use of unbiased language and organic communication about controversial subjects to aid comprehension. According to Werder [53] educative campaigns are most effective as a PR strategy when organizations use them consistently over a lengthy time period to drive behavioural change. This consistent approach makes the strategy more credible in the public sphere as foundations are laid for future learning. Additionally, they can be useful for stating a problem or threats to organizational goals and establishing confidence that once a problem is known, it can be resolved [54].

A facilitative strategy was accomplished by making resources available to the public so as to promote predisposed actions which in this context was conceptualized positive vaccine behaviour. A facilitative PR strategy is most effective when targeted at individuals who are already eager to be vaccinated, for instance, to improve vaccination rates. The study provides evidence that the GHS offered directions for members of the public to get easy access to mobile vaccination sites within communities. According to Zaltman [54], facilitative communication is useful when addressing challenges that belong to the shared consciousness of an organization and its public. In such cases, there is an agreement on the specific remedy to be implemented and there is openness to external assistance. Facilitative strategies may be an effective way for healthcare organizations in similar contexts to promote greater public awareness of the existence of various support schemes for achieving both organizational and client goals. Also, healthcare organizations may employ facilitative PR strategies to motivate individuals who are amenable to behavioural change because, without the incentive, these persons may lack the resource capacity required to contribute to organizational goals [16].

In many ways, the PR campaign followed by the GHS can be described as an exercise in persuasion [55]. According to Werder [48], a persuasive strategy derives its significance by appealing to shared values or emotions in the external environment. This strategy as used by the GHS emphasized a tailored presentation of facts to invoke public sympathy and concerted action. Persuasive PR managers are noted for the use of language that is not objective but goes the extra mile to show the cross-cutting significance of a matter at hand to compel initiative [56, 57]. Persuasive strategies and communication used by the service were often directive, containing an implicit/explicit call to action. According to Zaltman [54], “persuasive strategies are useful in cases where threats to organizational goals are not evident or considered essential by an organization’s publics, and as a result, commitment falls below expectation because the proposed organizational solution is in doubt.” Usually persuasive public relations strategies complement other PR strategies very well in contexts where publics are expected to take voluntary steps and the organization has no control through the management of resources valued by the public such as salaries [53, 57].

Officials also recognized that a high rate of illiteracy among the populace could be both a setback and an advantage [55, 57]. This high rate of illiteracy becomes a problem when developing a persuasive PR strategy because for a health organization with numerous experts who could speak convincingly about technical issues, a public with a high need for cognition would be an advantage when developing a PR strategy [58]. The fundamental idea of the elaboration likelihood model of persuasion is that when a group has a high appetite for mental exertion and craves cognition, the processing of communication will mostly take place along a central route [57]. The desired changes in attitudes and behaviour follow from deliberate and conscious processing of the arguments spelt out in the communication and as a result, such newly informed behaviour is likely to be persistent [59]. Future patterns of behaviour for individuals with an appetite for complex and detailed information are highly predictable as a result of the extensive elaboration [55, 57] and these individuals believe that they convinced themselves and were not convinced by the originator of the message. However, when faced with a large rural population and a high rate of illiteracy, it is safe to conclude that elaboration likelihood is low and the processing of communication must follow a peripheral route which requires little mental exertion. For these population groups, the GHS had to concentrate the communication using peripheral cues such as the credibility of the messenger and cues [57, 60]. Other healthcare organizations have used a similar approach in the country [61]. It follows from these mechanisms that behaviours acquired through the latter route are mostly not hinged on the presentation of more superior quality arguments, are temporary, and are not as revealing of subsequent sentiment as those formed using the central route [57].

## Conclusions

This is the first work to report on PR strategies used to combat vaccine hesitancy based on an identifiable and practical public relations process model [22, 23, 52]. Using the Hazleton and Long's (1988) public relations process model [22], we have successfully identified and documented PR strategies used by healthcare professionals to address COVID-19-related vaccine hesitancy in the country as a combination of informative, facilitative, persuasive, motivational and coercive PR strategies. Based on the framework used, we have not only been able to clarify an amorphous reality but also point out the practical advantages and potential limitations of the PR strategies identified from a strategic planning perspective [21, 53, 57]. These PR strategies were deployed by the GHS across both traditional and emerging/social media networks and have been largely efficacious and beneficial in combating COVID-19 vaccine hesitancy in Ghana.

### Strengths and limitations

This report is the first to document experiences of using PR strategies to achieve organizational goals in the context of public health management in sub-Saharan Africa. The case study design approach facilitated a more nuanced appreciation of the organizational context under investigation and exposed important themes. However, it also presented a number of limitations. Including interviews from regions which were known epicentres of infection in the study could have helped us to demonstrate concretely that the findings of the study are applicable to the contextual scenarios pointed out. However, as the Service operates a highly centralized administrative structure where regional and district offices only implement strategies determined from the top hierarchy, it is unlikely that our findings would alter significantly. Also, it was initially impossible to quantify the exact contribution of individual strategies employed to the overall outcome. However, some data on the level of vaccine hesitancy in the jurisdiction have now been published [9, 49, 50]. It was also unclear whether the findings applied to other vaccine scenarios beyond COVID-19 vaccines. More importantly, further studies would be required to clarify our understanding of potential confounding variables within the target population and the impact of global healthcare organizations such as the World Health Organization and the Global Alliance for Vaccines on the study’s findings.

### Implications for policy, practice and future research

Our work will inform public health policy, practice and research on the subject. The GHS will likely benefit from entrenching these PR strategies into an institutional culture and sustaining same in order to guarantee positive behaviour towards vaccination in Ghana in the future. The strategies are recommended for healthcare managers to prevent vaccine hesitancy in similar contexts. While these PR strategies may be useful for improving vaccine behaviour, or at least forestalling a crisis of vaccine hesitancy, they may also be applicable to other organizational goals. Quantitative research approaches are required to determine the extent to which these PR strategies are practised and correlated to the attainment of organizational targets.

## Supplementary Information

**Additional file 1:** **S1 Appendix A:** Interview guide for in-depth interviews.

**Additional file 2:** **S2 Appendix B:** Data file.

## Data Availability

All data generated or analysed during this study are included in this published article and its supplementary information files (Additional file 2: S2 Appendix B.xlsx: Data file).

## Abbreviations

AhR: Ahafo region
AIDS: Acquired immune deficiency syndrome
AR: Ashanti region
BR: Bono region
CIM: Chartered Institute of Marketing
CIPR: Chartered Institute of Public Relations
COREQ: Consolidated criteria for reporting qualitative research
COVID-19: Coronavirus disease
EI: Executive instrument
ELM: Elaborated Likelihood Model
ER: Eastern Region
GHS: Ghana Health Service
HIV: Human immunodeficiency virus
ICT: Information and Communication Technology
MOH: Ministry of Health
MP: Member of Parliament
NFC: Need-for cognition
PNDCL: Provisional National Defense Council Law
PR: Public relations
SARS CoV: Severe acute respiratory syndrome coronavirus
WHO: World Health Organization

## References

[1] Asare EO, Al-Mamun MA, Armah GE, Lopman BA, Parashar UD, Binka F (2020). Modeling of rotavirus transmission dynamics and impact of vaccination in Ghana. Vaccine.

[2] Vodicka E, Nonvignon J, Antwi-Agyei KO, Bawa J, Clark A, Pecenka C (2022). The projected cost-effectiveness and budget impact of HPV vaccine introduction in Ghana. Vaccine.

[3] Lartey BL, Damanka S, Dennis FE, Enweronu-Laryea CC, Addo-Yobo E, Ansong D (2018). Rotavirus strain distribution in Ghana pre-and post-rotavirus vaccine introduction. Vaccine.

[4] Enweronu-Laryea CC, Armah G, Sagoe KW, Ansong D, Addo-Yobo E, Diamenu SK (2018). Sustained impact of rotavirus vaccine introduction on rotavirus gastroenteritis hospitalizations in children< 5 years of age, Ghana, 2009–2016. Vaccine.

[5] Renner LA, Usuf E, Mohammed NI, Ansong D, Dankwah T, Kusah JT (2019). Hospital-based surveillance for pediatric bacterial meningitis in the era of the 13-valent pneumococcal conjugate vaccine in Ghana. Clin Infect Dis.

[6] Afolabi MO, Wariri O, Saidu Y, Otu A, Omoleke SA, Ebenso B (2021). Tracking the uptake and trajectory of COVID-19 vaccination coverage in 15 West African countries: an interim analysis. BMJ Glob Health.

[7] Amponsa-Achiano K, Frimpong JA, Barradas D, Bandoh DA, Kenu E (2022). Leveraging lessons learned from yellow fever and polio immunization campaigns during COVID-19 pandemic, Ghana, 2021. Emerg Infect Dis.

[8] Murphy J, Vallières F, Bentall RP, Shevlin M, McBride O, Hartman TK (2021). Psychological characteristics associated with COVID-19 vaccine hesitancy and resistance in Ireland and the United Kingdom. Nat Commun.

[9] Acheampong T, Akorsikumah EA, Osae-Kwapong J, Khalid M, Appiah A, Amuasi JH (2021). Examining vaccine hesitancy in Sub-Saharan Africa: a survey of the knowledge and attitudes among adults to receive COVID-19 vaccines in Ghana. Vaccines.

[10] Smith A, Yarwood J, Salisbury DM (2007). Tracking mothers’ attitudes to MMR immunisation 1996–2006. Vaccine.

[11] Sanche S, Lin YT, Xu C, Romero-Severson E, Hengartner N, Ke R (2020). High contagiousness and rapid spread of severe acute respiratory syndrome coronavirus 2. J Emerg Infect Dis.

[12] DeRoo SS, Pudalov NJ, Fu LY (2020). Planning for a COVID-19 vaccination program. JAMA.

[13] Omer SB, Salmon DA, Orenstein WA, Dehart MP, Halsey N (2009). Vaccine refusal, mandatory immunization, and the risks of vaccine-preventable diseases. N Engl J Med.

[14] Andre FE, Booy R, Bock HL, Clemens J, Datta SK, John TJ (2008). Vaccination greatly reduces disease, disability, death and inequity worldwide. J Bull World Health Org.

[15] Dib F, Mayaud P, Chauvin P, Launay O (2021). Online mis/disinformation and vaccine hesitancy in the era of COVID-19: Why we need an eHealth literacy revolution. Hum Vaccines Immunotherap..

[16] Wilson SL, Wiysonge C (2020). Social media and vaccine hesitancy. BMJ Glob Health.

[17] Trogen B, Oshinsky D, Caplan A (2020). Adverse consequences of rushing a SARS-CoV-2 vaccine: implications for public trust. JAMA.

[18] Machingaidze S, Wiysonge CS (2021). Understanding COVID-19 vaccine hesitancy. Nat Med.

[19] Khamsi R (2020). If a coronavirus vaccine arrives, can the world make enough. J Nat.

[20] Puri N, Coomes EA, Haghbayan H, Gunaratne K (2020). Social media and vaccine hesitancy: new updates for the era of COVID-19 and globalized infectious diseases. Human Vaccines Immunotherap.

[21] Smith RD (2020). Strategic planning for public relations.

[22] Hazleton V, Long LW (1988). Concepts for public relations education, research, and practice: a communication point of view. Commun Stud.

[23] Botan CH, Hazleton V (2010). Public relations theory II.

[24] Zaltman G, Duncan R (1977). Strategies for planned change.

[25] Abitbol A (2017). Examining the influence of public relations strategies over Facebook on student attitude. Public Relations J.

[26] Donkoh ET, Dassah ET, Owusu-Dabo E (2022). Optimization of a protocol for the evaluation of antibody responses to human papillomavirus (HPV) vaccination in low-resource settings. BMC Womens Health.

[27] Afrane YA. The COVID-19 situation in Ghana UK: RSTMH; 2021 https://rstmh.org/news-blog/news/the-covid-19-situation-in-ghana.

[28] Ghana becomes recipient of historic first shipment of COVAX vaccine [press release]. Online Statement: UN2021.

[29] 350,000 doses of COVID-19 vaccines have arrived in Ghana [press release]. Online Statement: UN2021.

[30] Ghana finds success in COVID-19 mass vaccination campaigns [press release]. Online statement: WHO, 29 April 2022 2022.

[31] Ghana Health Service. GHS Governance System Accra: Ghana Health Service; 2022 https://ghs.gov.gh/ghs-governance-system/.

[32] Palinkas LA, Horwitz SM, Green CA, Wisdom JP, Duan N, Hoagwood K (2015). Purposeful sampling for qualitative data collection and analysis in mixed method implementation research. Adm Policy Mental Health Serv Res.

[33] Etikan I, Musa SA, Alkassim RS (2016). Comparison of convenience sampling and purposive sampling. Am J Theor Appl Stat.

[34] Braun V, Clarke V (2006). Using thematic analysis in psychology. Qual Res Psychol.

[35] Alhojailan MI (2012). Thematic analysis: a critical review of its process and evaluation. West East J Soc Sci.

[36] Yıldırım A, Şimşek H (2013). Qualitative research methods in social sciences.

[37] Zamawe FC (2015). The implication of using NVivo software in qualitative data analysis: evidence-based reflections. Malawi Med J.

[38] Creswell JW, Poth CN. Qualitative inquiry and research design: Choosing among five approaches: Sage publications; 2016.

[39] Kornbluh M (2015). Combatting challenges to establishing trustworthiness in qualitative research. Qual Res Psychol.

[40] Treharne GJ, Riggs DW (2014). Ensuring quality in qualitative research. Qual Res Clin Health Psychol.

[41] Guest G, MacQueen KM, Namey EE (2012). Validity and reliability (credibility and dependability) in qualitative research and data analysis. Appl Thematic Anal.

[42] Tong A, Sainsbury P, Craig J (2007). Consolidated criteria for reporting qualitative research (COREQ): a 32-item checklist for interviews and focus groups. Int J Qual Health Care.

[43] Sarkodie B, Asiedu-Bekoe F, Laryea DO, Ampofo WK, Phillips RO, Samba A (2021). Overview of preparedness and response to COVID-19 in Ghana. Ghana Med J.

[44] Ghana Health Service. Greater Accra Region. Kindly visit a vaccination centre in your vicinity for your COVID-19 Vaccine. In: @_GHSofficial, editor. 11:12 am ed: Ghana Health Service; 2021.

[45] Bam NE (2021). Understanding the motivations for covid-19 vaccine hesitancy in South Africa: narrative literature review. Gender Behav.

[46] Tafuri S, Gallone MS, Cappelli MG, Martinelli D, Prato R, Germinario C (2014). Addressing the anti-vaccination movement and the role of HCWs. Vaccine.

[47] Nicholson A, Minicucci C, Liao J, editors. A Systems Approach to Increasing Vaccine Confidence and Uptake: Opportunities for Community-Based Strategies. The Critical Public Health Value of Vaccines: Tackling Issues of Access and Hesitancy: Proceedings of a Workshop; 2021: National Academies Press (US). 34351726

[48] Werder KP (2005). An empirical analysis of the influence of perceived attributes of publics on public relations strategy use and effectiveness. J Public Relat Res.

[49] Alhassan RK, Aberese-Ako M, Doegah PT, Immurana M, Dalaba MA, Manyeh AK (2021). COVID-19 vaccine hesitancy among the adult population in Ghana: evidence from a pre-vaccination rollout survey. Trop Med Health.

[50] Botwe B, Antwi W, Adusei J, Mayeden R, Akudjedu T, Sule S (2022). COVID-19 vaccine hesitancy concerns: findings from a Ghana clinical radiography workforce survey. Radiography.

[51] Kabakama S, Konje ET, Dinga JN, Kishamawe C, Morhason-Bello I, Hayombe P (2022). Commentary on COVID-19 vaccine hesitancy in sub-Saharan Africa. Trop Med Infect Dis.

[52] Hazleton V (2006). Toward a theory of public relations. Public Relat Theory.

[53] Werder KP. A theoretical framework for strategic communication messaging. The Routledge handbook of strategic communication. 2015:269–84.

[54] Zaltman G (1979). Knowledge utilization as planned social change. Knowledge.

[55] Petty RE, Cacioppo JT (1986). The elaboration likelihood model of persuasion. Communication and persuasion.

[56] Moran MB, Lucas M, Everhart K, Morgan A, Prickett E (2016). What makes anti-vaccine websites persuasive? A content analysis of techniques used by anti-vaccine websites to engender anti-vaccine sentiment. J Commun Healthcare.

[57] O’Keefe DJ. The elaboration likelihood model. The Sage handbook of persuasion: Developments in theory practice. 2013:137–49.

[58] Kitchen PJ, Kerr G, Schultz DE, McColl R, Pals H (2014). The elaboration likelihood model: review, critique and research agenda. Eur J Market.

[59] Haugtvedt CP, Petty RE. Need for cognition and attitude persistence. ACR North American Advances. 1989.

[60] Ghana Health Service. Traditional and Religious leaders are key stakeholders in our drive to get everyone vaccinated and protected from COVID-19. @_ghsofficial and @unicefghana have met with His Majesty Otumfuo Osei Tutu II, Asante Hene, and various chiefs of the Ashanti Region as well as Religious leaders in the Ashanti Region to sensitize them and solicit their support in the ongoing Covid-19 Vaccination Campaign. In: @_GHSofficial, editor.: twitter.com; 2022.

[61] CCIH. Christian Health Association of Ghana Engages Faith Community in Vaccine Outreach Virginia, USA: CCIH; 2021 [cited 2022 November, 16].

