# Supplementary material for: Public relations strategies employed by the Ghana Health Service to address COVID-19 vaccine hesitancy: a qualitative inquiry
Source: Trop Med Health. 2023 May 12;51:26. doi: 10.1186/s41182-023-00519-7 (PMC10175053; doi:10.1186/s41182-023-00519-7)
Supplement: Supplementary file 1 — Additional file 1: S1 Appendix A: Interview guide for in-depth interviews. [file 41182_2023_519_MOESM1_ESM.docx]

**Appendix 1: DATA COLLECTION INSTRUMENT**

KWAME NKRUMAH UNIVERSITY OF SCIENCE AND TECHNOLOGY

DEPARTMENT OF POLITICAL SCIENCE

The researcher is a student of the Department of History and Political Studies, Kwame Nkrumah University of Science and Technology (KNUST) researching the ***Public relations strategies employed by healthcare organizations to address vaccine hesitancy****.* The researcher would like to seek your candid views about this topic as an expert working in a healthcare organization. Hopefully, your feedback will help others to understand the contextual practice of public relations in healthcare settings and trigger potential skills transfer to other similar contexts. All answers will be treated as confidential and will be used for statistical analysis and research purposes.

**Interview guide**

***You may type in your responses in the relevant fields***

| First name: | Date: |
| --- | --- |
| Gender: | Venue: |
| Official designation: | Contact number/email: |
| Highest qualification (optional): |  |

1. Recently, the Ghana Health Service (GHS) launched a strategy to vaccinate all adults in Ghana for COVID-19. Do you consider vaccine hesitancy to be a threat to the achievement of vaccine-related strategic targets set by your organization? [*Hint: You may want to provide a more detailed explanation of your initial response*]
2. Do you consider the threat of vaccine hesitancy to the achievement of targets set by your organization a challenge that can be solved by public relations (PR) strategy? [*Hint: You may want to provide a more detailed explanation of your initial response*]
3. Can you describe some of the public relations strategies implemented by your organization to ward off the threat of vaccine hesitancy and to improve attitudes toward vaccinations? [*Hint: You may want to provide a more detailed explanation of your initial response*]
4. What would you say has been the impact of the available PR options listed above? [*Hint*: *You may want to take each strategy and comment on how much impact you feel has been generated*]
5. Can you share with me some of the contextual factors that can account for the success or failure of these PR strategies? [*Hint: This is a very important question and as much information as possible would be appreciated. You may want to take some time to reflect before providing a detailed response*.]
